# Arthritis of large joints shown as a rare clinical feature of cytokine release syndrome after chimeric antigen receptor T cell therapy: A case report

**DOI:** 10.1097/MD.0000000000010455

**Published:** 2018-04-20

**Authors:** Li-Xin Wang, Xiaoping Chen, Mingming Jia, Shengdian Wang, Jianliang Shen

**Affiliations:** a Department of Hematology, Navy General Hospital of PLA; b Key Laboratory of Infection and Immunity, Institute of Biophysics, Chinese Academy of Sciences, Beijing, China.

**Keywords:** arthritis, chimeric antigen receptor (CAR)-T cell therapy, cytokine release syndrome

## Abstract

**Rationale:**

Chimeric antigen receptor (CAR)-T cell therapy is a novel type of therapy that is being used in an increasing number of patients with acute lymphoblastic leukemia (ALL). Cytokine release syndrome (CRS) is the most common complication following CAR-T treatment, but the current understanding of the clinical manifestations and pathogenesis of CRS is still limited.

**Patient concerns:**

A 34-year-old male patient was diagnosed with ALL in June 2015. Complete remission (CR) was achieved after induction chemotherapy. The patient received 8 cycles of consolidation chemotherapy to maintain CR. In May 2017, the patient had recurrent ALL. Induction chemotherapy was given again, but without remission. In October 2017, CAR-T cell therapy was given. On October 14, the patient was pretreated with an FC regimen (fludarabine phosphate 50 mg qd on days 1–3; cyclophosphamide 0.4 g qd on days 1–3). CAR-T cells were infused on October 19 and October 20, with the number of infused cells at 2 × 10^5^/kg and 1 × 10^5^/kg, respectively. On October 25, the patient had a high fever, swelling, and pain in the large joints of the limbs, and joint effusion.

**Diagnosis:**

This patient was diagnosed with relapsed ALL, and he developed CRS after CAR-T therapy.

**Interventions:**

Tacilizumab (400 mg) was infused after CRS was diagnosed, and another dose of tacilizumab (240 mg) was given 6 days later. The pain was also treated with an analgesic drug. Methylprednisolone (1 mg/kg) was given to treat arthritis of the large joints.

**Outcomes:**

The patient's temperature was back to normal within 1 hour following the treatment of tacilizumab, but the pain in the large joints was progressively aggravated. The joint swelling and pain were obviously alleviated after the treatment of methylprednisolone, and the joint mobility was gradually recovered.

**Lessons:**

CRS after CAR-T therapy can manifest as a high fever with swelling and pain in the large joints of the limbs, similar to rheumatoid arthritis. Tocilizumab can lower the body temperature, but it has no significant effect on arthritis. Glucocorticoids can rapidly alleviate joint swelling and pain.

## 1 Introduction

In recent years, chimeric antigen receptor (CAR)-T cell therapy, a promising new cellular immunotherapy, has become a new option for the treatment of hematologic malignancies.^[1]^ At present, 2 CAR-T products have been approved for the treatment of acute lymphocyte leukemia (ALL) and non-Hodgkin lymphoma, meanwhile, a large number of clinical trials are underway to investigate the safety and efficacy of CAR-T in the treatment of other malignancies.^[2,3]^ Cytokine release syndrome (CRS) is the most common complication after CAR-T treatment, and it usually manifests as fever, fatigue, hypoxemia, and hypotension; severe cases can be fatal.^[4]^ We used CAR-T cells to treat a patient with relapsed ALL. He developed CRS manifesting as swelling and pain of multiple large joints, which was a rare clinical symptom.

## 2 Case report

**Figure 1:**
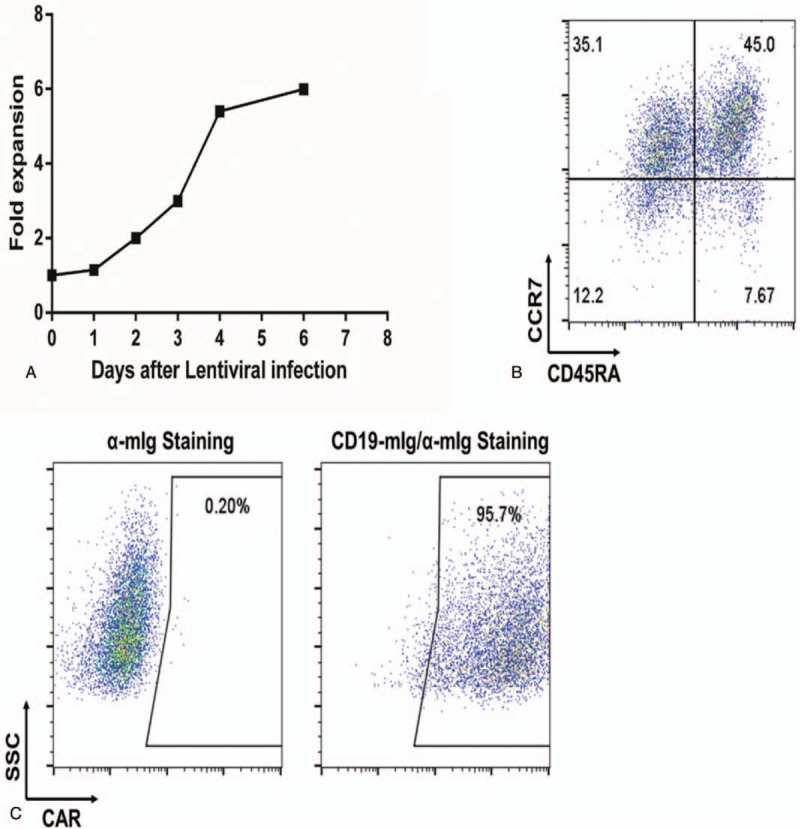
Gene transduction of αCD19 CAR and in vitro expansion of primary T cells. A, In vitro expansion of T cells following activation with αCD3/αCD28 coated magnetic beads on day 1 and transduction of CAR-encoding lentiviral vector on day 0. B, Representative FACS plots showing the proportion of CCR7+/CD45RA+ T cells (stem memory T cells). C, αCD19-specific CAR surface expression in T cells. The data shown in (B) and (C) were examined 5 days following transduction with CAR-encoding lentiviral vector. CAR = chimeric antigen receptor, FACS = fluorescence activated cell sorting.

**Figure 2:**
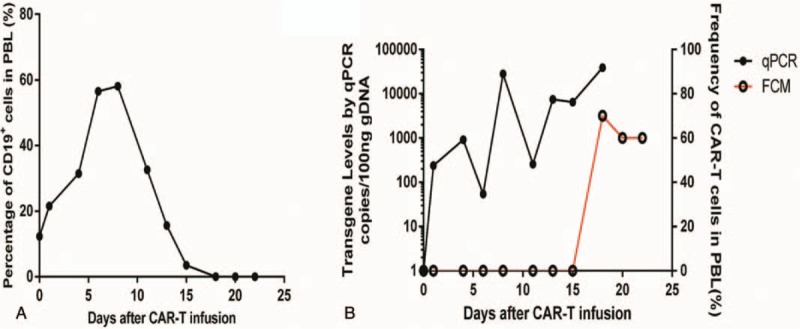
Cellular kinetic profiles of CD19^+^ cells and CAR-T cells in the blood after CD19 CAR-T cell infusion. A, Percentage of CD19^+^ cells in the blood on the indicated days after CAR-T cell infusion. B, CAR-T cell persistence in the blood as integrated transgene copies per 100 ng DNA measured by qPCR or frequency in the blood measured by flow cytometry. CAR = chimeric antigen receptor, qPCR = real-time quantitative polymerase chain reaction.

**Figure 3:**
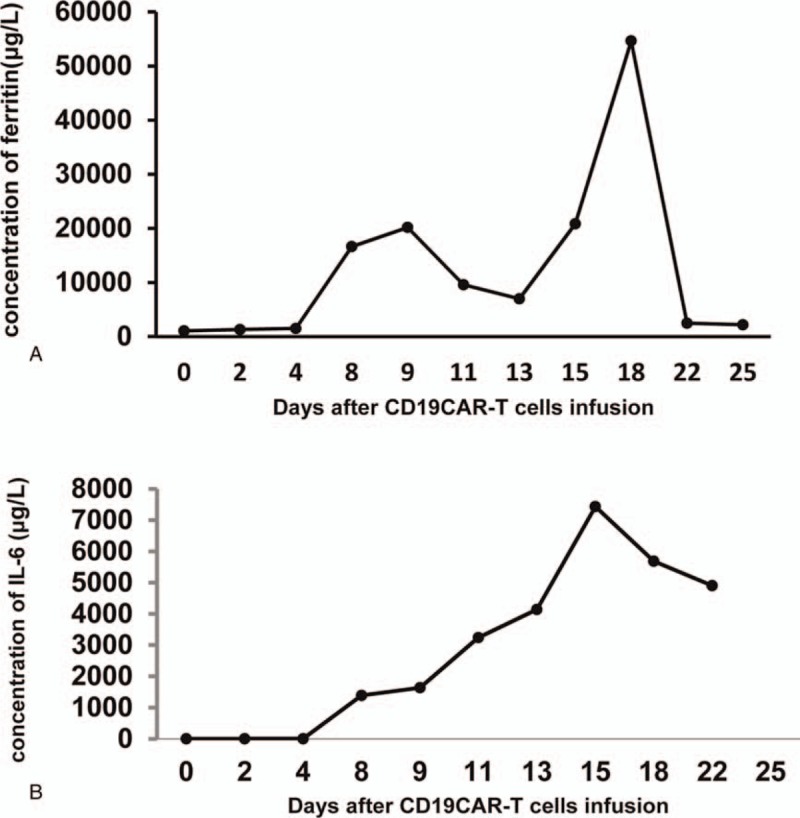
Dynamic changes of serum IL-6 and ferritin. A, Concentration of serum IL-6. B, Concentration of ferritin.

A 34-year-old male patient was diagnosed with acute lymphoblastic leukemia in June 2015. The bone marrow morphology showed 93% lymphoblasts and prolymphocytes. The bone marrow immunophenotype showed that the primitive region cells accounted for 36.2% of the nucleated cells. CD19, CD10, and HLA-DR were completely expressed and cCD79a was partially expressed. cMPO, cCD3, CD7, CD117, CD14, CD64, CD11c, CD15, CD34, CD16, CD13, CD11b, CD20, CD4, CD56, and CD33 were not expressed and E2A-PBX1 was positive. On July 2, 2015, VTCLP induction chemotherapy was administered (vincristine 2 mg on days 1, 8, 15, and 22; pirarubicin 40 mg on days 1–3; cyclophosphamide 1.26 g; and pegaspargase 4200 U, twice after a 14-day interval; Prednisone 55 mg on days 1–14, with a dose reduced on day 15). After treatment, a bone marrow examination showed complete remission (CR). On August 9, 2015, a consolidation therapy CAM regimen was given (cyclophosphamide 1.2 g on days 1 and 8; cytarabine 160 mg on days 1–3 and 8–10; 6-mercaptopurine 100 mg on days 1–7). Eight cycles of consolidation therapy were given between September 2015 and June 2016. The bone marrow biopsies performed during that period all indicated CR. Then, the patient was given a maintenance treatment of oral methotrexate and 6-mercaptopurine. In May 2017, the patient had right hip pain again and low fever without obvious causes. On June 1, 2017, the bone marrow biopsy showed 24% lymphoblasts and prolymphocytes, suggesting leukemia relapse. On June 3, HyperCVAD A chemotherapy was administered (cyclophosphamide 530 mg q/12 hours on days 1–3; mesna 1100 mg on days 1–3; vincristine 2 mg on days 4 and 11; perarubicin 88 mg on day 4; dexamethasone 40 mg on days 1–4 and 11–14). On June 30, a bone marrow examination showed CR. A hyper CVAD B regimen (methotrexate 1750 mg on day 1; cytarabine 1.75 g q/12 hours on days 2–5) was given during July 2017. Lumbar intrathecal chemotherapy was performed 12 times during the treatment period; the cerebrospinal fluid examination showed no abnormality, and the last intrathecal injection was in July 2017. On September 23, 2017, a bone marrow biopsy showed 90% lymphoblasts and prolymphocytes, suggesting leukemia relapse. On September 28, the patient was admitted into our hospital and enrolled in a clinical trial to be treated with CAR-T cell. Informed consent was obtained from the patient for the purpose of publication of this case report. The protocol was approved by the Human Ethics Committees of the Chinese Navy General Hospital. This trial was registered at ClinicalTrials.gov (NCT03281551). On October 11, the peripheral blood mononuclear cells were collected and subjected to CAR-T cell culture and amplification. Between October 14 and October 16, the patient was pretreated with an FC regimen (fludarabine phosphate 50 mg qd on days 1–3; cyclophosphamide 0.4 g qd on days 1–3). Then, 2 × 10^5^/kg and 1 × 10^5^/kg of CAR-T cells were respectively infused on October 19 and October 20. The frequency of CD19^+^ CAR-T in the product was 95.9%. The infused CAR-T cells were dominantly CD45RA^+^CCR7^+^ and CD45RA^-^CCR7^+^ phenotypes (Fig. 1). After the cell infusion, the CD19+ cell and CAR-T cell in peripheral blood were monitored by flow cytometry and real-time quantitative polymerase chain reaction (qPCR) (Fig. 2). The copy number of CAR gene detected by qPCR showed the continuous expansion of CAR-T cells. But CAR-T cells were not detected in blood by flow cytometry until day 15 after cell infusion. However, the frequency of CAR-T cells in peripheral blood lymphocytes (PBL) reaches 70%, suggesting a violent expansion of CAR-T cells in these 2 days. Then CAR-T cells remained 60% of PBL. The ratio of CD19+ cells in PBL detected by flow cytometry began to decrease on day 10 after cell infusion, and became undetectable on day 18, when the CAR-T expansion reached its peak. On October 23 the patient began to show muscle soreness. A pain killer was given, but with poor effect. On the early morning of October 25, the patient had a fever of 38.9°C, and it was accompanied by bilateral wrist, hip, knee, and ankle joint swelling and pain. The pain was especially excruciating in the right wrist, both knees, and the left ankle. The patient was treated with imipenem, an anti-inflammatory medication, but the temperature did not fall. Based on the evidence below: fever occurred 5 days after CAR-T cells infusion, highly increased serum level of IL-6, multiple arthritis, the patient was diagnosed as CRS. On October 26, tacilizumab (400 mg) was infused and the temperature was back to normal within 1 hour, which confirmed the CRS diagnosis. The pain in the knees, ankles, and wrists was progressively aggravated. The patient was unable to move his knee and ankle joints. An ultrasound indicated effusion in the large joints. Oral administration of oxycodone sustained-release tablets and external use of fentanyl patches and diclofenac diethylamine emulgel did not lead to any improvement. On October 28, the pain was alleviated with continuous intravenous analgesic drugs (hydromorphone and sufentanil), but the swelling was still there and the left ankle joint swelling continued to worsen. Another dose of tacilizumab (240 mg) was given on November 01, and on November 03, blood tests showed that: IgG was 3.06 g/L, IgA was 0.28 g/L, and IgM was 0.06 g/L, all of which were significantly decreased. Then, immunoglobulin was given. During the night of November 4, the patient had another fever of 38.6°C, and pulse oxygen saturation of 90%. The acute arterial blood gas analysis showed: PO_2_ 54 mm Hg, PCO_2_ 34 mm Hg, pH 7.53, and Lac 2.8 mmol /L, suggesting type 1 respiratory failure. Imipenem and cotrimoxazole were given, as well as ethylprednisolone (1 mg/kg). The bedside chest radiograph showed multiple lung infections. On November 7, a chest computerized tomography scan was taken and it showed bilateral multiple lung lesions (multiple patches and strips of blurry shadows). The antibiotics were changed from cotrimoxazole to amphotericin B, which is an antifungal. The joint pain was alleviated, and the knees and ankles were moveable after methylprednisolone treatment. During the process of clinical treatment, we monitored the level of plasma ferritin and IL-6 (Fig. 3), which were significantly increased after CAR-T cell infusion but decreased after tocilizumab treatment. On November 12, the patient was diagnosed as severe pulmonary infection with wheezing, coughing, and expectorating dark red bloody sputum. The situation became progressively worse. At 8:00 pm, the patient had a sudden increase in difficulty breathing, which was persistent, and he died.

## 3 Discussion

CRS is the most common complication of CAR-T cell therapy.^[5]^ In this case, the relapsed ALL patient developed a high fever 3 days after CAR-T infusion, and multijoint swelling and pain on day 5. Based on the clinical signs and increased serum IL-6 and ferritin, he was diagnosed as CRS and treated with tocilizumab. His temperature dropped to normal within 1 hour following the tocilizumab treatment. The diagnosis of CRS was confirmed; however, the joint swelling and pain were not improved with the decrease of the body temperature. On the contrary, the joint swelling and pain were further aggravated, and there was fluid accumulated in the joint cavity. The joint swelling and pain were improved after cortisol treatment, which is consistent with the characteristics of noninflammatory arthritis. According to the literature, CRS typically manifests with fever, malaise, anorexia, and myalgia. Any system in the body can be affected, including the cardiovascular, respiratory, integumentary, gastrointestinal, hepatic, renal, hematological, and nervous systems.^[4]^ But the serious arthritis symptoms were rarely reported in patients with CAR-T therapy. The current case presented a rare clinical feature, characterized by multiple forms of arthritis, which was controlled by a corticosteroid rather than tocilizumab.

## Author contributions

**Conceptualization:** Li-Xin Wang.

**Data curation:** Xiaoping Chen.

**Investigation:** Li-Xin Wang, Mingming Jia, Shengdian Wang, Jianliang Shen.

**Supervision:** Li-Xin Wang, Jianliang Shen.

**Writing – original draft:** Li-Xin Wang.

**Writing – review and editing:** Li-Xin Wang, Shengdian Wang.
