# Arthritis of large joints shown as a rare clinical feature of cytokine release syndrome after chimeric antigen receptor T cell therapy: A case report: Erratum

**DOI:** 10.1097/MD.0000000000011341

**Published:** 2018-06-22

In the article, “Arthritis of large joints shown as a rare clinical feature of cytokine release syndrome after chimeric antigen receptor T cell therapy: A case report”,^[1]^ which appeared in Volume 97, Issue 16 of *Medicine*, tocilizumab was misspelled throughout the article and appeared incorrectly as tacilizumab.
